# A Model for Predicting Cervical Cancer Using Machine Learning Algorithms

**DOI:** 10.3390/s22114132

**Published:** 2022-05-29

**Authors:** Naif Al Mudawi, Abdulwahab Alazeb

**Affiliations:** Department of Computer Science, College of Computer Science and Information System, Najran University, Najran 55461, Saudi Arabia; afalazeb@nu.edu.sa

**Keywords:** machine learning (ML), cervical cancer, human papillomavirus (HPV), gradient boosting, support vector machine (SVM)

## Abstract

A growing number of individuals and organizations are turning to machine learning (ML) and deep learning (DL) to analyze massive amounts of data and produce actionable insights. Predicting the early stages of serious illnesses using ML-based schemes, including cancer, kidney failure, and heart attacks, is becoming increasingly common in medical practice. Cervical cancer is one of the most frequent diseases among women, and early diagnosis could be a possible solution for preventing this cancer. Thus, this study presents an astute way to predict cervical cancer with ML algorithms. Research dataset, data pre-processing, predictive model selection (PMS), and pseudo-code are the four phases of the proposed research technique. The PMS section reports experiments with a range of classic machine learning methods, including decision tree (DT), logistic regression (LR), support vector machine (SVM), K-nearest neighbors algorithm (KNN), adaptive boosting, gradient boosting, random forest, and XGBoost. In terms of cervical cancer prediction, the highest classification score of 100% is achieved with random forest (RF), decision tree (DT), adaptive boosting, and gradient boosting algorithms. In contrast, 99% accuracy has been found with SVM. The computational complexity of classic machine learning techniques is computed to assess the efficacy of the models. In addition, 132 Saudi Arabian volunteers were polled as part of this study to learn their thoughts about computer-assisted cervical cancer prediction, to focus attention on the human papillomavirus (HPV).

## 1. Introduction

Human life is plagued with difficulties because it is difficult to predict when problems arise. In general, women usually experience several difficulties in their lifetime. One of the most critical ailments they may face is cervical cancer, which causes many problems [1]. The elevated mortality age of uterine cancer is due to women’s lack of knowledge about the importance of early detection [2]. Cervical cancer is a dangerous cancer, which threatens women’s health worldwide, and its early signs are relatively difficult to detect [3]. It is responsible for damaging deep tissues of the cervix and can gradually reach other areas of the human body, such as the lungs, liver, and vagina, which can increase the difficulties involved [4]. However, while cervical cancer is a slow-growing malignancy, precancerous advances have made early detection, prevention, and therapy possible. Cervical cancer has been reduced in most nations over past decades as detection technologies have improved. This year, 4290 people are predicted to die from cervical cancer [5]. The fatality rate has dropped by roughly half since the mid-1970s, thanks in part to enhanced screening, which has resulted in the early identification of cervical cancer. The death rate has reduced from over 4% per year in 1996–2003 to less than 1% in 2009–2018 [6]. The pre-invasive stages of cervical cancer of the uterus last for a long time. Screening tests can provide successful treatment of precancerous-stage lesions, so that cancer can be prevented. Nonetheless, it has been determined that the death rate in underdeveloped nations is exceptionally high, since they do not benefit from state-provided preventive strategies, such as free immunization programs and national assessment programs.

When the cervix’s human papillomavirus (HPV) infection is left untreated, cervical cancer develops [7]. Because it causes neoplastic development, the human papillomavirus (HPV) is the most common infectious agent in cervical cancer. The improper proliferation of cervical cancer cells and the multiplication of abnormal cells as a result of a malignant phase is referred to as neoplastic progression [8]. The healthcare industry regularly generates massive amounts of data that can be used to extract information for forecasting future sickness based on a patient’s treatment history and health data. Furthermore, these areas can be enhanced by leveraging crucial data in healthcare. Machine learning helps individuals process vast amounts of complex medical data in healthcare and then analyze it for therapeutic insights. Doctors can then use this information to provide medical care. As a result, patient satisfaction can be improved when machine learning (ML) is employed in healthcare.

Cervical cancer is one of the most common malignancies among women worldwide. Recently, many studies have been conducted on cervical cancer using modern techniques that provide prediction in the early stage. Using machine learning has contributed to early prediction [9]. Therefore, the most important causes of this disease among female populations are lack of awareness, lack of access to resources and medical centers, and the expense of undergoing regular examination in some countries [10]. Machine learning has improved the performance of analyses and the generation of accurate patient data. One researcher [11] employed text mining, machine learning, and econometric tools to determine which core and enhanced quality attributes and emotions are more relevant in forecasting clients’ satisfaction in different service scenarios. Their paper presents findings related to health product marketing and services, and proposes an automated and machine-learning-based technique for generating insights. It also aids healthcare/health product e-commerce managers improve the design and execution of e-commerce services. Moreover, the importance of continuous quality improvement in the performance of machine learning algorithms from a health care management and management information technologies point of view is demonstrated in this paper by describing different kinds of machine learning algorithms and analyzing healthcare data utilizing machine learning algorithms [12]. This study identified algorithms that are better suited for the categorization of negative and positive cervical cancer for clinical use. Cervical cancer can be diagnosed with the help of such algorithms. Deep learning has shown a significant impact on health and medical imaging, which helps evaluate the diagnostic accuracy of deep learning (DL) algorithms in identifying pathologies in medical imaging [13].

The objectives of this study are as follows:

- To analyze and classify cervical cancer using machine learning algorithms that will help doctors accurately diagnose the cancer.
- To identify the correlations between the parameters that are likely to be responsible for cervical cancer.
- To conduct a survey that identifies women’s concerns about cervical cancer, and provides a message to the readers as well as the research community.

Section 2 provides a literature review, Section 3 describes the research methodology, and Section 4 includes the results and discussion.

## 2. Literature Review

This section provides the literature selection criteria (LSC) and the papers that have been collected to review the literature from all the databases. The literature selection criteria (LSC) section shows how we selected related papers based on the selection criteria, after collecting the articles from the databases. Looking at papers published between 2010 and November 2020, this research has explored several electronic databases, such as Institute of Electrical and Electronics Engineers (IEEE) Xplore, PubMed, National Center for Biotechnology Information (NCBI), Springer, Google Scholar, and Elsevier. Based on the selected articles, the literature review is provided in detail below.

### Literature Selection Criteria 

The advantage of selection criteria is that it is possible to work according to a plan, especially when downloading the papers. According to the time duration set, articles can be searched, and fake journals can be skipped. In terms of search criteria, the research paper must be a conference paper or journal article, and it must use a machine-learning-based model or program intended solely for cervical cancer prediction. In addition, the following conditions must be met:

- Purposes must be included in the research paper.
- The time frame being surveyed is from 2010 to 30 November 2021. It is important to analyse the previous studies’ insights
- We do not include any research work that has not yet been printed, or is not peer reviewed.

In [14], the authors conducted a survey-based study on cervical cancer detection, including performance analysis to determine the accuracy of various distinctive types of architecture in an artificial neural network (ANN), where the ANN was used for identifying cancerous, normal, and abnormal cells. The authors of [15] used cervigram images to illustrate a method of screening cervical cancer with the oriented local histogram technique (OLHT), which can increase edges, and the dual-tree complex wavelet transform (DT-CWT), which can help achieve multi-resolution images. Using a UCI data repository and six machine learning (ML) classifiers, ref. [16] proposed a model that can predict the exact level of cervix infection. Data pre-processing was carried out with physician verification to extract some features and to perform validation. To complete the study, 10-fold cross-validation is utilized to assess the performance of the suggested model. Another key study was published in [16], which used machine learning classifiers (SVM, QUEST, C&R tree, and MLP). The investigation examined distinct metrics such as accuracy, sensitivity, specificity, and area under the curve (AUC). The QUEST parameters were 95.55%, 90.48%, 100%, and 95.20%, respectively. This research proposed a federated learning method for machinery malfunction diagnostics to address the data island problem. Each participant’s model training is implemented on a local level, and a self-supervised learning scheme is provided to improve learning performance [17].

Five different machine learning algorithms are used by [18], including random forest, KNN, C5.0, SVM, and RPart. After finishing the training and evaluating the performance of all of the classifiers (C5.0, RF, RPART, SVM, and KNN), the best options in terms of accuracy were investigated, showing values of 97%, 96.9%, 96%, 88%, and 88%. Machine learning (ML) algorithms such as decision tree, random forest, and logistic regression were used in conjunction with the voting model. In [19], cervical cancer was detected using a dataset containing four target parameters (biopsy, cytology, Schiller, and Hinselmann), as well as 32 risk factors, collected from the University of California (UCI). Machine learning (ML) algorithms were applied, including the the decision tree and decision jungle approaches. The study observed that the decision tree algorithm showed a higher value (98.5%). In another study using the Microsoft Azure ML tool, an appropriate data mining technique was developed from the boosted decision tree, decision forest, and decision jungle algorithms to detect cervical cancer [20]. The models’ performances were measured in terms of accuracy, area under the receiver operating characteristic (AUROC) curve, specificity, and sensitivity, with 10-fold cross-validation applied to the outputs to improve the decision tree algorithm’s performance, reaching a value of 97.8% on the AUROC curve. The authors of [21] presented a survey-based study on cervical cancer prevention from the perspective of women in Bug, IRI, and Mayuge in Eastern Uganda, using a questionnaire to collect data from 900 women aged 25 to 49 years. After measuring and scoring the women’s knowledge and statements about cervical cancer treatment, the data was analyzed using Stata 12.0 software. After doing bivariate and multivariate analysis, the authors discovered that 794 women, or roughly 88.2%, had heard of the condition. A majority of 557 women (70.2%) acquired their information from the radio, while a minority of 120 women (15.1%) got their information from health care organizations.

The authors of [22] analyzed various machine learning approaches used from 2006 to 2017 to diagnose cervical cancer. In this research, a comparison was made using existing relevant works based on cervical cancer medical data, to determine the benefits and drawbacks of different approaches. Most studies had used unbalanced medical image datasets. The survey also mentioned employing deep learning to predict cervical cancer. Furthermore, the goal of [23] was to see how well the Cox proportional hazard regression model and the deep learning neural network model predicted survival in cervical cancer patients. A dataset from the University of California, Irvine, was used in the study [23], which included age, number of pregnancies, contraceptive use, smoking habits, and chronological records of sexually transmitted infections (STDs). The study’s essential purpose was to use Hinslemann screening methods to predict cervical cancer. With 10-fold validation, a data mining strategy was used with the boosted decision tree, decision forest, and decision jungle approaches. Moreover, on the AUROC (area under receiver operating characteristic) curve, the boosted decision tree method achieved a forecast precision of 98%. The best example of using electronic health record (EHR) data to predict cervical cancer is [24]. Four machine learning classifiers were used to predict cancer. The random forest algorithm produced the best results, with an AUC (area under the curve) of 0.97 one day before diagnosis, up from 0.70 a year before diagnosis. The primary purpose of [25] was to create a method that can anticipate the early effects of radiation on bone metastases in cervical cancer patients. The researchers employed class imbalance learning (CIL) in data mining to tackle the challenge of an imbalanced dataset. To deal with the issue of imbalanced data categorization, many models, such as ant-miner, RIPPER, Ridor, PART, ADTree, C4.5, ELM, and weighted ELM, with the synthetic minority over-sampling approach (SMOTE) were used. The study aimed to assist in the early detection of cervical cancer. The study showed the use of machine learning in defining a data validation mechanism to improve the performance of cervical cancer prediction. The study also suggested genetic assistance as an optional strategy to enhance the validity of the prediction. Additionally, [26] has presented a method based on machine learning approaches for identifying cardiac disease. Classification algorithms were used to construct the system. The model suggested a conditional mutual information feature selection method to overcome the feature selection problem. Feature selection methods are utilized to improve classification accuracy and shorten the time it takes to develop a classification system.

Furthermore, the fundamental purpose of [27] was to examine how big data analytics and machine-learning-based approaches may be used for diabetes. The results demonstrate that the proposed machine-learning-based system might score as high as 86% on the diagnostic accuracy of DL. Health specialists and other stakeholders collaborated to create classification models that would assist in diabetes prediction and the design of prevention measures. Based on the findings, the authors review the literature on machine models and propose an intelligent framework for diabetes prediction. Anther study has been conducted [28] where a methodology for heart disease was developed using the UCI repository dataset and healthcare monitors to estimate the public’s risk of heart disease. In addition, classification algorithms were employed to classify patient data to detect cardiac disease, such as doosted decision tree and decision forest. The classification was performed using data from the benchmark dataset during the training phase. At the testing stage, accurate patient data was used to determine whether illness existed. The results demonstrate that the proposed model based on machine learning could score as high as 92% on the diagnostic accuracy of DL. Comparative analysis of existing research are provided in Table 1.

Based on the above review, it can be stated that several traditional algorithms have been used to predict cervical cancer; still, the models do not achieve a satisfactory level, because the selection of important features is the most crucial part of machine learning, and the authors have not specified how the chosen features were selected. In addition, just using traditional deep learning algorithms does not indicate that a model is suitable for practical implementation in the healthcare sector; rather, model customization is required to remove the overfitting and make it faster for a clinical application. Nonetheless, this research has come up with an effective solution. Various state-of-the-art techniques are used in this study to take this research to a satisfactory level and assist doctors in diagnosing cervical disease.

## 3. Methodology

**Figure 1:**
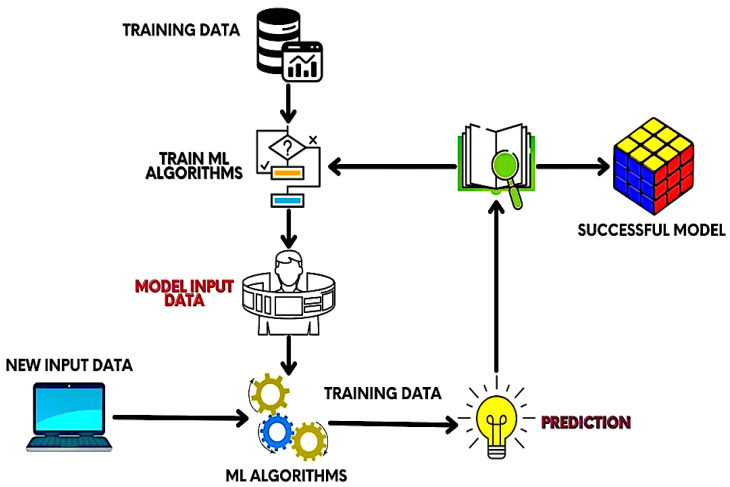
Proposed research model for classifying cervical cancer.

The proposed research methodology is classified into several segments: research dataset, data preprocessing, predictive model selection (PMS), and training method. Figure 1 depicts an architectural diagram of the proposed research; by looking at Figure 1, it can be clearly observed that the architectural diagram has been separated into four phases, because the model presented in this research performs some essential tasks in each stage. Details on research data collection are described in the Research Dataset section. The Data Preprocessing section mentions how to remove noise from the dataset and make it useful for feeding in machine learning. The type of predictive model selected to predict cervical cancer in this research is shown in the PMS portion. The requisites for model training are shown in the Training Methods section. Finally, we design the platform to provide an overall pipeline of cervical cancer prediction using the Python programming language. This research implements an algorithm that is better suited for the categorization of negative and positive cervical cancer diagnoses for clinical use. Cervical cancer can be diagnosed with the help of algorithms including decision tree, logistic regression, support vector machine (SVM), K-nearest neighbours (KNN), adaptive boosting, dradient boosting, random forest, and XGBoost. The sequence and consequences are presented in the following sections.

The proposed ML-based model is depicted in Figure 1. The training data will be fed to the system at the beginning of the model training. Then, ML algorithms are adopted. After that, model input data and new input data are applied to the scheme to train the architecture properly. Finally, prediction is performed on the newly accumulated data.

### 3.1. Research Dataset

The UCI repository contributed to the dataset “Cervical Cancer Risk Factors for Biopsy” [29]. The collection contains information about 858 people’s activities, demographics, and medical history. Multiple missing values occur in this dataset for hospital patients as a result of several patients declining to answer questions due to privacy concerns [30]. The collection has 858 instances, each with 32 properties. The dataset includes 32 variables and the histories of 858 female patients [30]. The dataset includes 32 variables and the histories of 858 female patients, including factors such as age, IUD, smokes, STDs, and so on. The research dataset’s attributes are provided in Table 2.

### 3.2. Data Preprocessing

Data preprocessing is divided into three sections, which are as follows: data cleaning, data transformation, and data reduction. Data preprocessing is critical since it directly impacts project success. Data impurity occurs when attributes or attribute values contain noise or outliers, and redundant or missing data [30]. We have removed the missing values and outliers from this dataset. The data transformation stage is kept in place to change the data into suitable forms for the mining process. This research combines normalization, attribute selection, discretization, and concept hierarchy generation. When dealing with a huge amount of data, analysis becomes more difficult when the data dimension is large. The data reduction approach is employed in this research to overcome this. It seeks to improve storage efficiency, while lowering the cost of data storage and processing. We have applied the dimension reduction technique because it is another useful technique that can be used to mitigate overfitting in machine learning models. For that, we have applied the principal component analysis (PCA) technique.

### 3.3. Predictive Model Selection (PMS)

Several machine learning classification algorithms have been used in the PMS, namely support vector machine (SVM), decision tree classifier (DTC), random forest (RF), logistic regression (LR), gradient boosting (GB), XGBoost, adaptive boosting (AB), and K-nearest neighbor (KNN). This section has highlighted some of the algorithms that have achieved a satisfactory level of accuracy on the adopted research dataset. Thus, we have illustrated the theoretical interpretation of these algorithms in the following subsections.

#### 3.3.1. Decision Tree (Dt)

Both classification and regression problems can be solved with the classification and regression tree or CART algorithm, which is also called the DT. The DT looks a lot like the branches of a tree, which is why the word ‘tree’ is included in its name. The decision tree starts from the ‘root node’ just as the tree starts from the root. From the root node, the branches of this tree spread through different decision conditions; such nodes are called decision nodes (and called leaf nodes after making a final decision).

#### 3.3.2. Random Forest (Rf)

Ensemble learning enhances model performance by using multiple learners. RF is also a kind of ensemble learning. Following the RF bagging method reduces the chances of results being affected by outliers. This works well for both categorical and continuous data. Datasets do not need to be scaled, and the higher the number of learners, the more computational resources are required for complex models. In this algorithm, the decision is made by voting. Such an algorithm is called ensemble learning. Random forests are made up of many trees or shrubs. Just as there are many trees in the forest, random forests also have many decision trees. The decision that most trees make is considered the final decision.

#### 3.3.3. Adaptive Boosting (AB)

The adaptive boosting technique creates a powerful learner by combining the knowledge of a number of weak learners. In this scenario, every single weak learner utilizes the exact same input, often known as a training set. Every initial input or piece of training data is given the same amount of importance. The responsibility for correcting the incorrect predictions made by the first weak learner is passed on to the next weak learner, who is given greater weight on the predictions made by the first weak learner and is turned over to the next weak learner. As a result, the errors that the second weak learner made in its predictions are passed on to the following weak learner in the same fashion, but with increased weight. The same process is continued until the number of inaccurate forecasts is reduced to a manageable level. In the end, a powerful learner is developed via the combined efforts of all the weak learners. In this way, the amount of inaccuracy in the forecast is reduced.

#### 3.3.4. Support Vector Machine (SVM)

The support vector machine algorithm can be used for classification and regression problems. However, SVMs are quite popular for relatively complex types of small or medium classification datasets. In this algorithm, data points are separated by a hyperplane, and the kernel determines what the hyperplane will look like. If we plot multiple variables in a normal scatter plot, in many cases, that plot cannot separate two or more data classes. The kernel of an SVM is a significant element, which can convert lower-dimensional data into higher-dimensional space, and thus differentiate between types [31]. The following equations are used in the case of SVM (1) and (2) [32]:

(1) $$\vec{w}\cdot \vec{x}+b=0$$

In this case, w is the (possibly normalized) average vector to the hyperplane. These two specific hyperplanes bound the “margin” in the region or area, and the maximum hyperplane lies halfway between them. These hyperplanes can be defined by equations using a normalized or standardized dataset.

$$\mathrm{Plus}-\mathrm{plane}\;=\vec{w}\cdot \vec{x}+b=0$$

$$\mathrm{Minus}-\mathrm{plane}\;=\vec{w}\cdot \vec{x}-b=0$$

Therefore, the width or the margin of the two hyperplanes for data classification can be written as follows:

(2) $$width=\frac{\vec{W}}{abs\left(\vec{W}\right)}$$

### 3.4. Radial Basis Function (RBF) Kernel Support Vector Machine (SVM)

The support vector machine (SVM) performs well on linear and nonlinear data. This method of classifying nonlinear data includes the radial base function. Putting data in the function space relies heavily on the kernel function [33]. When plotting many variables in a typical scatter plot, it is often impossible to distinguish between various sets of data. An SVM’s kernel is a technique for transforming lower-dimensional input into higher-dimensional space and identifying different classes. In addition, the radial basis function is a nonlinear function. The support vector machine’s most popular feature is its ability to classify objects automatically. Infinite-dimensional space can be mapped to any input with this kernel.

(3) $$K\left(x_{1},x_{2}\right)=\exp\left(-\frac{{\left|x_{1}-x_{2}\right|}^{2}}{2\sigma^{2}}\right)$$

After utilizing Equation (1), we can obtain the following:

(4) $$f(X)=\sum_{i}^{N}\alpha_{i}y_{i}k\left(X_{i},X\right)+b$$

By applying Equation (3) in (4), we get a new function, where *N* represents the trained data.

(5) $$f(X)=\sum_{i}^{N}\alpha_{i}y_{i}\exp\left(-\frac{{\left|x_{1}-x_{2}\right|}^{2}}{2\sigma^{2}}\right)+b$$

#### Gradient Boosting

The gradient boosting algorithm also follows the sequential ensemble learning method. Through loss optimization, weak learners gradually become better than previous weak learners. For example, the second weak learner is better than the first, the third weak learner is better than the second, and so on. As the weak learner periodicity increases, the amount of error in the model decreases, and the model becomes a stronger learner. The gradient boosting algorithm works relatively well for regression-type problems [34].

The difference between gradient boosting and adaptive boosting is that in adaptive boosting, error is gradually reduced by updating the weight of the wrong predictive samples. In gradient boosting, the loss function is optimized, and each loss is optimized [35]. The amount of error also decreases. To optimize this loss function, each weak learner changes its alternative weak learner model, so that the next weak learner is better than the previous one. Gradient boosting consists of three components: weak learner, loss function optimization, and additive model. The following Equations (6)–(11) show the working procedure of the gradient boosting algorithm mathematically [36]:

1. “Reconfigure the function estimate with a constant value”
  (6) $$\hat{f}(x)={\hat{f}}_{0},{\hat{f}}_{0}=\gamma ,\gamma \in \mathbb{R},{\hat{f}}_{0}=\underset{\gamma }{\arg\min}\sum_{i=1}^{n}L\left(y_{i},\gamma \right)$$
2. “For each iteration “t = 1,…,T”:”
  (7) $$\begin{array}{c} \mathrm{Compute}\;\mathrm{pseudo}-\mathrm{residuals}\;r_{t},r_{it}=-{\left[\frac{\partial L\left(y_{i},f\left(x_{i}\right)\right)}{\partial f\left(x_{i}\right)}\right]}_{f\left(x\right)=\hat{f}\left(x\right)}\\ \;\mathrm{for}\;i=1,\ldots ,n \end{array}$$

Include the latest function $g_{t}$ (*x*) (it can be any model, but here we are applying decision trees) as regression on pseudo-residuals.

(8) $$\;{\left\{\left(x_{i},r_{it}\right)\right\}}_{i=1,\ldots ,n}$$

“Determine optimal coefficient “*ρ*_*t*” at “*g*_*t* (*x*)” about the initial loss function”

(9) $$\rho_{t}=\underset{\rho }{\arg}\min\sum_{i=1}^{n}L\left(y_{i},\hat{f}\left(x_{i}\right)+\rho \cdot g_{t}\left(x_{i},\theta \right)\right)$$

“Improve current approximation”

(10) $$\begin{array}{cc} & \hat{f}(x)\mathrm{where}{\hat{f}}_{t}(x)=\rho_{t}\cdot g_{t}(x)\\ & \hat{f}(x)\leftarrow \hat{f}(x)+{\hat{f}}_{t}(x)=\sum_{i=0}^{t}{\hat{f}}_{i}(x) \end{array}$$

3. The ultimate GBM model will be the addition of the elementary constant and the entire following function update:
  (11) $$\hat{f}(x)=\sum_{i=0}^{T}\hat{f}(x)$$

## 4. Result Analysis 

This section is categorized into four parts: empirical consequence report (ECP), exploratory cervical data analysis (ECDA), computational complexity analysis (CCA), comparative analysis, and survey data analysis (SDA).

### 4.1. Empirical Consequence Report (ECP) 

The accuracy of predictions from the classification algorithms is estimated by applying a classification report. The report demonstrates the precision, recall, and f1 score of the key classification metrics on a per-class basis. By using true positive (TP), false positive (FP), true negative (TN), and false negative (FN), the metrics are computed [37]. Table 3 demonstrates the classification reports of the several traditional machine learning algorithms where the precision, recall, and F1 scores are denoted by “P”, “R”, and “F1”. Precision is the ratio of the model’s correct positive estimates to the total (correct and incorrect) positive estimates; recall is the ratio of being able to predict positive as positive; and F1 is the weighted average of precision and recall (this score considers both false positives and false negatives). A classification report has been included in the table, where 0 means negative class and 1 means positive class.

To obtain the classification report [38], the following Equations (12)–(15) are used.

P: The relationship between the accurate positive estimate generated by the model and the overall (correct and inaccurate) positive estimate. It is articulated as:

(12) $$P=\frac{TP}{TP+FN}\;\;$$

Recall/sensitivity: Positivity is represented by the ratio of accurate to inaccurate predictions. It is written in mathematical notation as follows:

(13) $$F2=2\cdot \frac{TP}{TP+FN}$$

F1: This is the harmonic mean of precision and recall, and it provides a more accurate estimate of the amount of misclassification cases than the accuracy metric. It can be expressed numerically as:

(14) $$F2=\frac{Precision\cdot Recall}{Precision+Recall}\;$$

Accuracy: It is the measure of all the instances correctly predicted. It is given as:

(15) $$\mathrm{Accuracy}=\frac{TP+TN}{TP+TN+FP+FN}$$

The mean squared error (MSE), mean absolute error (MAE), root mean squared error (RMSE), and R-squared (R2) are frequently used to measure a model’s effectiveness in terms of regression analysis. The accuracy of gradient boosting and XGBoost is obtained with the performance metrics, as shown in Table 3. The MAE illustrates the commonality of clearly distinguishing between specific and predicted values within the dataset. Similarly, the MAE shows the traditional square difference between main and anticipated standards. The RMSE also computes the standard deviation of the residuals. Finally, the R-squared (R2) represents the fraction of variation inside the variable quantity defined by the regression toward the mean model [38]. We have interpreted different algorithms with the corresponding evaluation matrices. From the finding in Table 3, the highest classification scores have been achieved with random forest (RF), decision tree (DT), and adaptive boosting. In contrast, XGBoost provides a higher level of regularization for the gradient boosting algorithm. Advanced regularization (L1 and L2) is utilized in XGBoost to increase model generalization. In terms of performance, XGBoost is superior to the gradient boosting algorithm. Its training is quite fast, and it may be dispersed across numerous clusters if necessary. Because we need to determine the distinction between a classification model, XGBoost, and gradient boosting, we have separated these models into a separate table (Table 4) to survey the accuracy measurements of each of them, and found the highest accuracy of 100 with gradient boosting.

### 4.2. Exploratory Cervical Data Analysis (ECDA)

**Figure 2:**
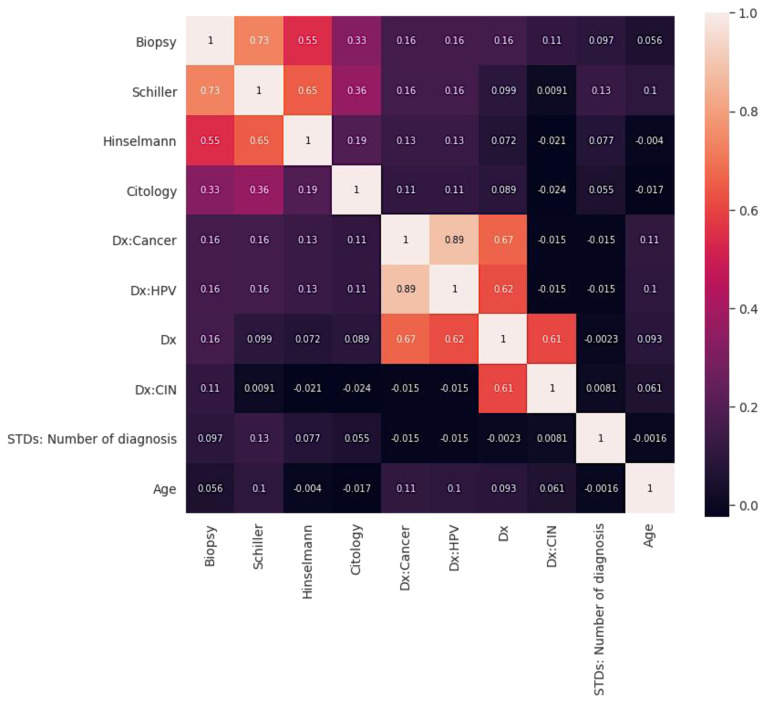
Correlations between different variables of cervical cancer.

Figure 2 shows the correlation graph. Correlation describes how two or more variables are connected [39]. These variables may be input data features used to forecast our target variable. Correlation is a mathematical method used to evaluate how one variable moves or shifts in relation to another. It informs us about the intensity of the relationship between the two variables. It is a bivariate analysis measure that defines the relationship between various variables [39]. Moreover, finding the correlation is significant in cervical analysis because essential factors can be identified by finding the relationship between each variable. Two characteristics (variables) may be positively correlated with one another. 

In the same way, two features (variables) can be negatively correlated with one another. This implies that as the value of one variable rises, the other variable(s) falls. On the other hand, if one variable’s value increases or decreases, but the value of the other variable(s) does not, this indicates no correlation. The correlations are illustrated in Figure 2.

**Figure 3:**
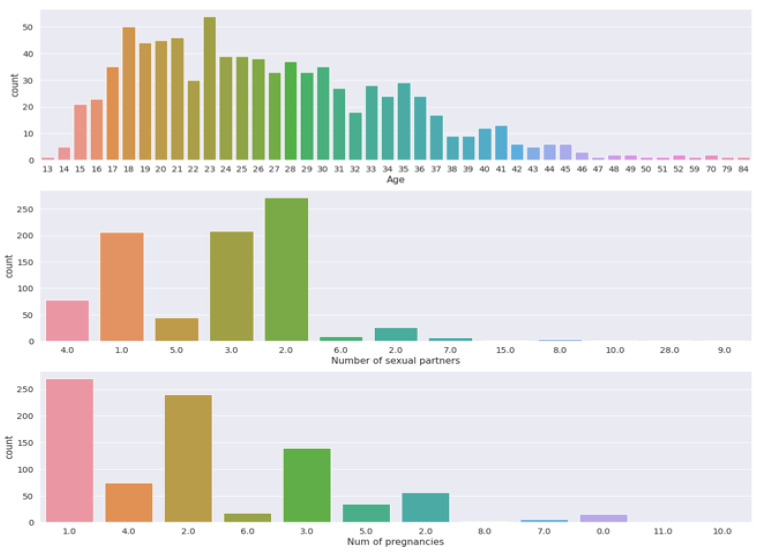
Count measurement in terms of the number of pregnancies, number of sexual partners, and age.

**Figure 4:**
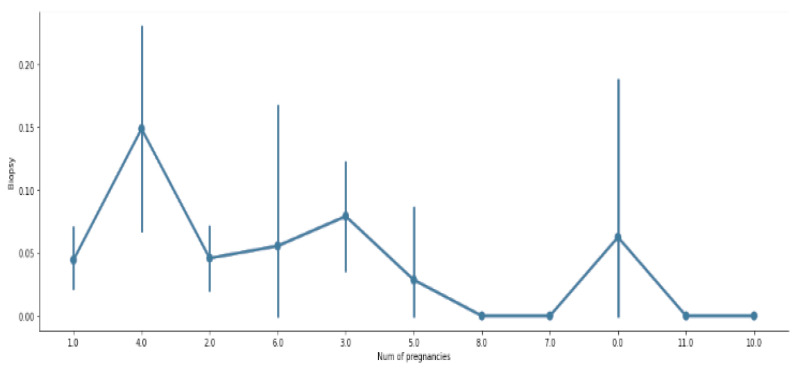
Visualization of comparison between biopsy and number of pregnancies.

Figure 3 and Figure 4 visualize the count measurement regarding the number of pregnancies, the number of sexual partners, and age, and a comparison between biopsy and number of pregnancies. The cervix is the uterus’s lower, narrowest portion. It creates a canal that leads to the vaginal opening. Cervical biopsies can be performed in a variety of ways. As shown in Figure 4, it is evident that a relationship between biopsy and pregnancy exists, but occasionally fluctuates. 

### 4.3. Computational Complexity Analysis (CCA) 

Machine learning computational complexity is a quantitative examination of the possibilities for effective computer learning [40]. It is focused on successful and general learning algorithms and works within recently deployed machine inference models based on computational complexity theory. We conducted a complexity analysis of various classic algorithms because these types of algorithms have previously been utilized to identify cervical cancer. Researchers confront numerous challenges regarding algorithm selection, so determining the computational complexity before creating a model is critical. Table 5 shows a short summary of different algorithms, indicating the complexity analysis of regression, dataset training, and prediction.

### 4.4. Validation

This research has applied cross-validation, which is a method that examines the research model to achieve better residuals [41]. The problem with validation is that it does not indicate how good data will be when it is used to make new estimates for a new result. The better solution to this problem is not applying the entire dataset when we run data training, which requires removing some of the data before training starts. Then, when we finish training with the data, we can use the data removed in the assessment to show how the model fits on “new’’ data. We have applied five-fold cross validation, and we did a resampling method that uses different portions of the data to test and train a model on various iterations. This model achieved satisfactory performance, and as the data size is not large, we aim to apply these validation indicators in the next phase as our research is still ongoing.

### 4.5. Survey Data Analysis (SDA)

Another part of our research is conducting survey data analysis. To determine how many people are aware of cervical cancer, we have completed survey questionnaires based on the aim of this research. In this research, a stratified sampling technique has been used; stratified sampling is a similar or homogenous group-based sampling method [42]. Our priority for this survey was to analyze the number of women who are less aware of cervical cancer. It is certainly true that many women often feel too shy to talk about the mentioned diseases with their parents, so in this research, we have highlighted this issue, so that essential steps can be taken to raise awareness. In addition, the core biopsy test is significant, and many are not familiar with this test. This was the primary reason for taking a survey and analyzing the data. All members of the same group usually have the same characteristics; such groups are called strata. Table 6 shows some major survey questions (number of responses: N = 132; 94.69% answered all questions correctly).

**Figure 5:**
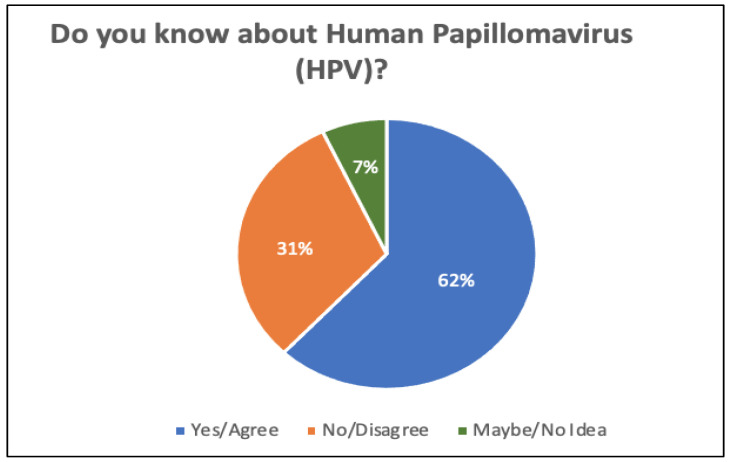
Number of responses regarding the awareness of human papillomavirus (HPV).

Figure 5 illustrates the number of responses in terms of awareness of human papillomavirus (HPV). By looking at Figure 5, it can be clearly seen that 31% of the participants are not aware of HPV, while 62% are aware of the virus. Only 7% of respondents were unsure. 

**Figure 6:**
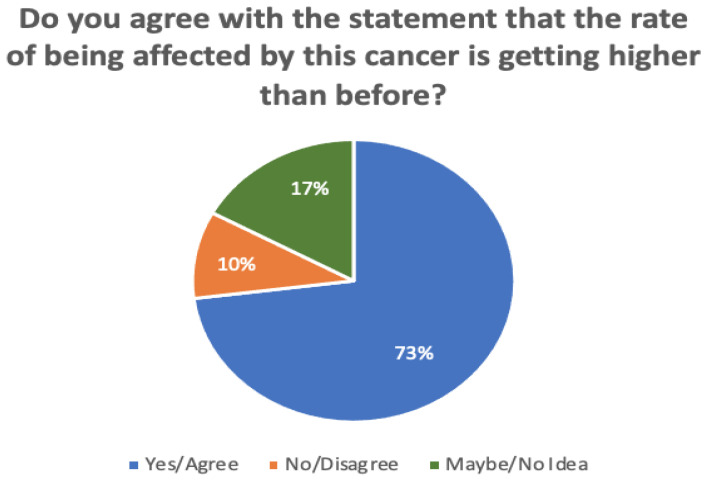
Survey responses regarding whether or not the rate of being affected by cervical cancer is becoming higher than before.

In addition, Figure 6 compares the responses in terms of whether or not the rate of being affected by cervical cancer is becoming higher than before. It is noticeable that 73% of participants agreed with this statement, while 17% disagreed. A minority of participants (around 10%) were unsure. 

**Figure 7:**
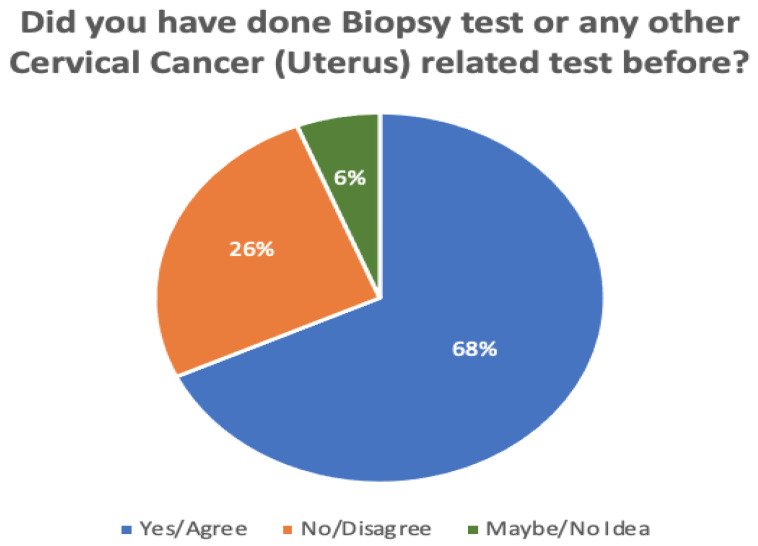
Total percentage of individuals who have undergone a biopsy test or another cervical cancer (uterus)-related test before.

**Figure 8:**
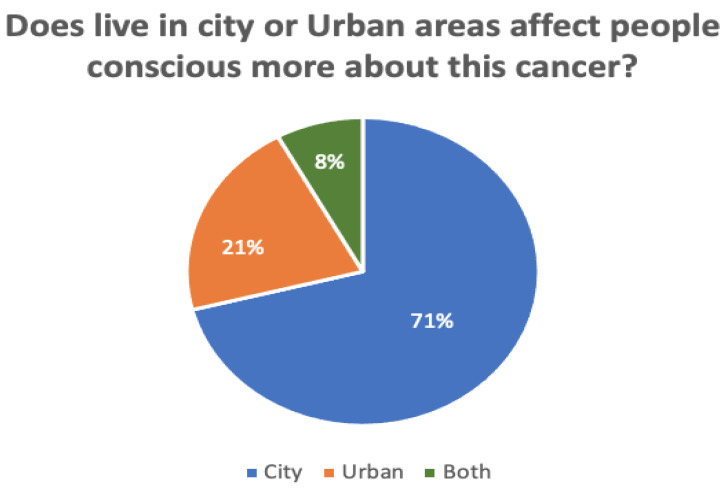
The awareness level in rural and urban areas regarding cervical cancer.

Figure 7 and Figure 8 compare the proportions of biopsy tests and awareness levels in rural vs. urban areas. A total of 132 responses were recorded during the survey. Of these, 26% of all participants had not yet undergone a biopsy test, while 6% of participants were unsure. According to the survey, those who live in cities are more aware (71%) than those in rural areas (21%). Another 8% of participants said both are equivalently aware of cervical cancer. 

## 5. Discussion 

Based on the findings of this research, it can be stated that the objectives of this paper have been achieved. Its research methodology was enriched with a set of algorithms including decision tree (DT), logistic regression (LR), support vector machine (SVM), K-nearest neighbors (KNN), adaptive boosting, gradient boosting, random forest (RF), and XGBoost. The research has reached a satisfactory result for both predictions and classification. This investigation also observed that the DT and RF algorithms were used in conjunction with the Microsoft Azure machine learning (ML) method to achieve a proper data mining technique for predicting cervical cancer. The study has further noticed that the performances of the traditional algorithms used in previous research are comparatively low. It is important to use data scaling, conduct missing value removal, and select a suitable algorithm in the case of disease analysis and prediction. Still, previous research has not shown the details of this pipeline. It is a matter of great concern that this work has not been accomplished much in previous research using gradient boosting algorithms. Since the gradient boosting algorithm also follows the sequential ensemble learning method, the wave learners gradually get better than their previous wave learners through this method of loss optimization. 

It is essential to point out that the researchers did not restrict their effort to simply developing the model; rather, they also validated and evaluated the model’s performance. Several validation strategies, including ROC-AUC, confusion matrix, and cross-validation, were applied by the researchers, and the researchers found that the efficacy with respect to cervical cancer is adequate. In addition, the current research investigated the most important predictors and the algorithms that are most frequently utilized for the purpose of cervical cancer prediction. During the preprocessing phase, some aspects of the patients’ samples, such as the length of time they drank alcohol and their HIV and HSV2 infection status, revealed that factors whose samples had undergone modest variations could not be considered accurate predictors. Fewer predictors may need to be analyzed in subsequent studies because of the potential importance of a given characteristic for the community or the patient’s social status. This may make it easier to conduct the research more quickly. However, with the help of this machine learning model, women have the opportunity to benefit from knowing more about cervical cancer and what effect it has on the human body. This study will focus on women in order to identify which symptoms or parameters are important for identifying for cervical cancer, as well as the causes and effects of these symptoms and parameters.

This study has further performed a survey with 132 participants in Saudi Arabia to explore cervical cancer awareness, focusing on the human papillomavirus (HPV). This data is mainly gathered to identify individuals’ thoughts and comments regarding HPV and cervical cancer. By conducting survey-based data analysis, the study has evaluated and rated the women’s awareness and behaviors regardings cervical cancer care. It is notable that the authors did not address why HPV is responsible for cervical cancer; also, the survey did not show how much women knew about the biopsy test. 

While working with the proposed models and algorithms, a number of limitations have been observed. First of all, the DT algorithm is very unstable, which means that a slight change in the data will significantly change the layout of the best decision tree. It is insufficiently reliable. With similar data, several other predictors perform better. Second, this study faced massive problems while dealing with the dataset, because numerous data have been enumerated and interpreted in the data pre-processing stage. The model will provide an optimum result only if a considerable number of data-processing techniques have been adopted. Third, the survey data have been preserved to apply machine learning to conduct sentiment analysis regarding cervical cancer, but in this study, the researchers could not accommodate different data-processing techniques to apply the ML models.

## 6. Conclusions

Early detection increases the likelihood of successful treatment in the pre-cancer and cancer stages. Being aware of any signs and symptoms of cervical cancer can also aid in avoiding diagnostic delays. This research has focused on cervical cancer using conventional machine learning (ML) principles and several traditional machine learning algorithms, such as decision tree (DT), logistic regression (LR), support vector machine (SVM), and K-nearest neighbors (KNN). In terms of cervical cancer prediction, the highest classification score of 100% has been achieved with the random forest (RF), decision tree (DT), adaptive boosting, and gradient boosting algorithms. In contrast, 99% accuracy has been found with SVM. The results of these algorithms are applied to identify the most relevant predictors. We have received satisfactory accuracy compared to the support vector machine algorithm. The findings of this study revealed that the SVM model could be used to find the most important predictors. As the number of essential predictors for analysis decreases, the computational cost of the proposed model decreases. The disease can be predicated more accurately with the use of machine learning. Furthermore, boosting patients’ personal health and socio-cultural status can lead to cervical cancer prevention.

In addition, this research conducted a survey in Saudi Arabia, with 250 participants, to learn their thoughts in response to the investigation of cervical cancer; risk factors have also been identified through some data analyses. In the future, this research will experiment with many datasets, analyze various deep learning algorithms and their computational complexity, and show a pipeline that can extract more important insights through statistical analysis in further research.

## Figures and Tables

**Table 1:** Comparative analysis of existing research.

Source	Used Dataset	Classifiers	Evaluation Matrix	Findings
[14]	UCL-858 patients and 36 attributes	ROC-AUC	ML method	Cervical cancer diagnosis
[15]	Patient demographics	N/A	Neural network	Applied Cox proportional techniques
[16]	UCL repository	ROC-AUC	Decision tree	Hinslemann screening methods
[17]	EHRs	AUC	Random forest	Traditional approaches
[18]	N/A	G-mean and F-measure	ADTree	Handling the data imbalance
[19]	Dataset collected from the University of California (UCI)	Using four target parameters: biopsy, cytology, Schiller, and Hinselmann, as well as 32 risk factors	Machine learning (ML) algorithms are applied, such as decision tree and decision jungle approaches.	Decision tree algorithm shows a higher value of 98.5%.
[20]	Data mining technique	(AUROC)	The Microsoft Azure ML tool	Decision tree algorithm, a higher value range of 97.8% on the AUROC curve.
[21]	A survey-based study on cervical cancer to collect data from 900 women aged 25 to 49 years	N/A	Using Stata 12.0 software.	A majority of 557 women (70.2%) acquired their information from the radio, while a minority of 120 women (15.1%) got their information from health care organizations.
[22]	Unbalanced medical image dataset	Assisted in determining cervical cancer, and benefits and drawbacks of different approaches	Machine learning approaches	Employing deep learning to predict cervical cancer with high probability.
[23]	A dataset from the University of California, Irvine	Used Hinslemann screening methods to forecast cervical cancer	Deep-learning neural network	Boosted decision tree, decision forest, and decision jungle approaches.
[24]	Electronic health record (EHR) data	Four machine learning classifiers	Random forest algorithm	The boosted decision tree method produced a precise forecast of 98%.
[25]	Data radiation on bone metastases in cervical cancer patients	Ant-miner, RIPPER, Ridor, PART, ADTree, C4.5, ELM, and Weighted ELM	Class imbalance learning (CIL)	Suggested genetic assistance as an optional strategy to enhance the validity of the prediction.
[26]	N/A	Classification algorithms are used to construct the system	Method based on machine learning approaches	Utilized to improve classification accuracy and shorten the time it takes to develop a classification system.
[27]	Data related to diabetes	Health specialists and other stakeholders collaborate	Big data analytics and machine-learning-based approaches may be used for diabetes.	Machine learning-based system might score as high as 86% on the diagnostic accuracy Of DL.
[28]	UCI repository dataset	Classify patient data to detect cardiac disease	Boosted decision tree, decision forest	Score as high as 92% on the diagnostic accuracy of DL.

**Table 2:** Attributes of the research dataset.

No.	Attribute	Type
1	Age	Int
2	Number of sexual partners	Int
3	First sexual intercourse	Int
4	Number of pregnancies	Int
5	Smokes	Bool
6	Smokes (years)	Bool
7	Smokes (pack/year)	Bool
8	Hormonal contraceptives	Bool
9	Hormonal contraceptives (years)	Int
10	IUD	Bool
11	IUD (years)	Int
12	STDs	Bool
13	STDs (number)	Int
14	STDs: condylomatosis	Bool
15	STDs: cervical condylomatosis	Bool
16	STDs: vaginal condylomatosis	Bool
17	STDs: vulvo-perineal condylomatosis	Bool
18	STDs: syphilis	Bool
19	STDs: pelvic inflammatory	Bool
20	STDs: genital herpes	Bool
21	STDs: molluscum contagiosum	Bool
22	STDs: AIDS	Bool
23	STDs: HIV	Bool
24	STDs: hepatitis B	Bool
25	STDs: HPV	Bool
26	STDs: number of diagnoses	Int
27	STDs: time since first diagnosis	Int
28	STDs: time since last diagnosis	Int
29	Dx: cancer	Bool
30	Dx: CIN	Bool
31	Dx: HPV	Bool
32	Dx	Bool

**Table 3:** Classification report of the machine learning algorithms for classifying cervical cancer.

Algorithm	For the Case of “0”				For the Case of “1”			
	Purpose	P	R	F1	P	R	F1	Accuracy Score
Logistic Regression	Cervical cancer prediction	0.98	1.00	0.99	1.00	0.77	0.87	0.98
SVM		0.99	1.00	1.00	1.00	0.92	0.96	0.99
Random Forest		1.00	1.00	1.00	1.00	1.00	1.00	1.00
Decision Tree		1.00	1.00	1.00	1.00	1.00	1.00	1.00
Adaptive Boosting		1.00	1.00	1.00	1.00	1.00	1.00	1.00
KNN		0.95	1.00	0.97	1.00	0.31	0.47	0.95

**Table 4:** Accuracy measurement of gradient boosting and XGradient boosting.

Algorithm	MAE	MSE	RMSE	Accuracy	R2
Gradient Boosting	7.330935195811098 × 10^−165^	0.0	0.0	1.00	1.00
XGBoost	0.04847228	0.021919228	0.14805144	0.68628035	0.68628035

**Table 5:** Computational complexity of machine learning algorithms.

Algorithm	Classification/Regression	Training	Prediction
Decision Tree	C + R	$O\left(n^{2}p\right)$	O(p)
Random Forest	C + R	$O\left(n^{2}pn_{\mathrm{trees}}\right)$	$O\left(pn_{\mathrm{trees}}\right)$
Gradient Boosting $(n_{\mathrm{trees}})$	C + R	$O\left(npn_{\mathrm{trees}}\right)$	$O\left(pn_{\mathrm{trees}}\right)$
SVM (Kernel)	C + R	$O\left(n^{2}p+n^{3}\right)$	$O\left(n_{sv}p\right)$
k-Nearest Neighbours	C + R	-	O(np)

**Table 6:** Some major survey questions for investigating cervical cancer.

Some Major Survey Questions that Match Survey Goal	Response N = 132		
	Yes/Agree	No/Disagree	Maybe/No Idea
Have you done a biopsy test or any other cervical cancer (uterus)-related test before?	68%	26%	6%
Is everyone in your family aware of cervical cancer?	76%	20%	4%
Do you agree with the statement that the rate of being affected by this cancer is becoming higher than before?	73%	10%	17%
Do you know about human papillomavirus (HPV)?	62%	31%	7%
Does living in a city or urban area affect how conscious people are of this cancer?	71%	21%	8%
Have you had a biopsy or any other cervical cancer (uterus)-related test before?	54%	35%	11%

## Data Availability

Data of Cervical cancer Availability Statement: dataset was obtained from the open-access Cervical cancer (Risk Factors) Data Set database of Cervical Cancer Risk Factors for Biopsy and are available at https://archive.ics.uci.edu/ml/datasets/Cervical+cancer+%28Risk+Factors%29 (accessed on 24 March 2022).
